# Correlation between immune suppressor cells and cytokines in the peripheral blood of patients with chronic lymphocytic leukemia

**DOI:** 10.3389/fonc.2026.1777390

**Published:** 2026-04-15

**Authors:** Aleksandr V. Ponomarev, Zinaida A. Sokolova, Yulia B. Chernykh, Tatiana E. Byalik, Irina Zh. Shubina, Pavel V. Tsarapaev, Marina V. Pikhovkina, Tatiana A. Mitina, Maria A. Baryshnikova, Vyacheslav S. Kosorukov

**Affiliations:** 1 FSBI «N.N. Blokhin National Medical Research Center of Oncology» оf the Ministry of Health of the Russian Federation, Moscow, Russia; 2 M.F. Vladimirsky Moscow Regional Research and Clinical Institute, Moscow, Russia; 3 I.M. Sechenov First Moscow State Medical University of the Ministry of Health of the Russian Federation (Sechenov University), Moscow, Russia

**Keywords:** CLL, correlation, cytokines, hypothesis, IDO, inflammation, MDSC, Treg

## Abstract

**Background:**

Patients with chronic lymphocytic leukemia (CLL) exhibit elevated levels of various cytokines and increased numbers of immunosuppressive cell subpopulations. However, the relationship between cytokines and suppressor cells remains unclear. Studies in this field may contribute to a better understanding of the role of cytokines in the accumulation of suppressor cells.

**Methods:**

Serum levels of 47 cytokines in patients with CLL and healthy donors were measured using multiplex analysis. The numbers of suppressor cells, including mMDSCs, Tregs, CD14+/IDO+ monocytes, and gMDSCs in peripheral blood, were determined using flow cytometry.

**Results:**

A significant difference in the levels of 29 cytokines was observed between primary CLL patients and healthy donors. A statistically significant increase in the numbers of mMDSCs, Tregs, and CD14+/IDO+ monocytes was observed in CLL patients compared to healthy donors, while no significant differences were noted in the CD15+/LOX-1+gMDSCs numbers between CLL patients and healthy donors. Correlations were found between the levels of several cytokines and immunosuppressive cell numbers. Among these, two cytokines, MIP-1α and M-CSF, showed a positive correlation with two suppressor cell subpopulations. Furthermore, the levels of these cytokines differed between patients with Binet stage B and stage C CLL.

**Conclusions:**

Our findings suggest that these two cytokines play a key role in the immunosuppression observed in CLL patients. These results, demonstrating a correlation between cytokine levels and the numbers of suppressor cells in CLL patients, are consistent with our previous hypothesis. This hypothesis states that the simultaneous action of growth factors and pro-inflammatory cytokines can lead to immune system inhibition.

## Introduction

Inflammation is a normal reaction of the body to an injury or infection. The body releases chemicals that trigger an immune response to fight the infection or to heal damaged tissue (1). At the same time, the hallmarks of cancer include evasion of immune surveillance and the process of pro-tumor inflammation (2, 3). It appears that in some cases inflammation can stimulate immune cells, while in other cases, such as a developing tumor, it can lead to the suppression of immunity. Despite a large number of studies on inflammation, the reasons for this dual role remain unclear. Earlier, we proposed a hypothesis to explain this apparent contradiction (4).

According to the hypothesis, immune activation at the site of inflammation requires that the levels of pro-inflammatory cytokines prevail over low quantities of growth factors. These conditions may be observed in acute inflammation. The synergistic effect of the simultaneous action of growth factors and pro-inflammatory cytokines is realized when a sufficient amount of growth factors combines with pro-inflammatory cytokines, which results in the suppression of monocytes/macrophages. Such a situation can occur in the context of chronic inflammation and cause immunosuppression.

Monoclonal B-lymphocytes accumulate both in the bone marrow and in the peripheral blood of patients with chronic lymphocytic leukemia (CLL). Significant immunosuppression develops as a concomitant process in CLL, which leads to an increased risk of infections and secondary neoplasms (5–7). Studies have reported increased populations of suppressor cells, such as monocytic myeloid-derived suppressor cells (mMDSCs), T regulatory cells (Tregs), CD14+/IDO+ monocytes (8), and HLA-DRlo/CD11b+/CD33+/CD14-/CD15+ granulocytic MDSCs (gMDSCs) (9), in the peripheral blood of patients with CLL. Various studies have shown that MDSCs (10, 11) and Tregs (12) have immunosuppressive function. A number of authors have noted that enhanced numbers of mMDSCs (13, 14), HLA-DRlo/CD11b+/CD33+/CD14-/CD15+ gMDSCs (9), and Tregs (15, 16) are associated with a worse prognosis in patients with CLL. Literature data have demonstrated that the peripheral blood of patients with CLL contains increased levels of various cytokines, including pro-inflammatory cytokines (17, 18), which may be indicators of inflammation. In addition, reports have provided evidence of increased levels of various angiogenic factors. There is no complete understanding of this phenomenon, since leukemia cells do not depend on a network of blood vessels to provide basic physiological functions (19).

Given the pleiotropic nature of various cytokines, it seems important to determine their potential role in immunosuppression, particularly in patients with CLL. To achieve this goal, it is necessary to study the relationship between these cytokines and suppressor cells. This will help to better understand the causes of immunosuppression and the complex immune interactions in CLL. The present study aimed to evaluate the correlation between serum cytokine levels and the number of immunosuppressive cells in the peripheral blood of patients with CLL.

## Materials and methods

### Patients

The study was performed at the N.N. Blokhin National Research Medical Center of Oncology and the M.F. Vladimirsky Moscow Regional Research and Clinical Institute from 09/2021 to 02/2024. Peripheral blood samples were collected from 38 primary patients with CLL and 4 patients with relapsed CLL prior to the start of therapy. The control group included 15 healthy donors.

### Flow cytometry

Peripheral blood was collected from patients with CLL and healthy donors into EDTA tubes. The analysis was performed on the day of collection. Peripheral blood mononuclear cells (PBMCs) were isolated using Ficoll-Paque Plus (Cytiva) to evaluate the populations of mMDSCs, CD14+/IDO+ cells, and Tregs. The gMDSC population was assessed in whole blood, and erythrocyte lysis was performed using the IOTest 3 Lysing Solution (Beckman Coulter). The cells were stained according to the manufacturer’s protocol using fluorochrome-conjugated antibodies, as described in **Supplementary Tables 1, 2**. The Foxp3 Staining Buffer Set (eBioscience) was used for intracellular detection of FOXP3, and the Intracellular Fixation & Permeabilization Buffer Set (eBioscience) was used for IDO protein detection, following the manufacturer’s instructions. Flow cytometry was performed on a FACS Canto II flow cytometer equipped with FACSDiva™ software (Becton Dickinson). Human BD Fc Block (BD Biosciences) was used to block non-specific binding. Cell clusters were excluded using FSC-A vs. FSC-H and SSC-A vs. SSC-H. The gating strategy is presented in the **Supplementary Materials**. BD™ CompBeads Anti-Mouse Ig, κ (BD Biosciences) were used for compensation setup.

### Multiplex cytokine analysis

The levels of the cytokines sCD40L, EGF, Eotaxin, FGF-2, FLT-3L, Fractalkine, G-CSF, GM-CSF, GROα, IFNα2, IFNγ, IL-1α, IL-1β, IL-1RA, IL-2, IL-3, IL-4, IL-5, IL-6, IL-7, IL-8, IL-9, IL-10, IL-12 (p40), IL-12 (p70), IL-13, IL-15, IL-17A, IL-17E/IL-25, IL-17F, IL-18, IL-22, IL-27, IP-10, MCP-1, MCP-3, M-CSF, MDC, MIG, MIP-1α, MIP-1β, PDGF-AA, PDGF-AB/BB, TGFα, TNFα, TNFβ, and VEGF-A were estimated in the serum of 35 primary CLL patients, 4 relapsed CLL patients, and 15 healthy donors using a multiplex system (Human Cytokine/Chemokine/Growth Factor Panel A HCYTA-60K-PX48, Merck) on a MagPix instrument (Merck) according to the manufacturer’s protocol. The results were processed with the Belysa^®^ Analysis Software.

### Statistics

Statistical analysis was performed using STATISTICA v.7, R v.4.0, and GraphPad Prism. Normality testing was conducted using the Shapiro–Wilk test. Continuous variables were expressed as medians and interquartile ranges: Me (Q1–Q3), as the data were not normally distributed. Differences between continuous variables were assessed using the Kruskal–Wallis test with Dunn’s *post hoc* test for comparisons among three groups, and the Mann–Whitney U test for comparisons between two groups. Univariate and multivariate analyses were performed to examine the relationships between cytokines and suppressor cells. Spearman’s rank correlation coefficient was calculated, and multivariate linear regression was conducted. To account for multiple comparisons in correlation analysis, adjustment was performed using the Benjamini–Hochberg false discovery rate (FDR) method. Prior to fitting the linear model, all data were ranked to mitigate the influence of outliers. Due to the biological interrelatedness of cytokines, multicollinearity was assessed by calculating the variance inflation factor (VIF). Only models with a VIF not exceeding 2 were considered. A two-tailed p-value < 0.05 was considered statistically significant for all analyses.

## Results

### Characteristics of the patients and healthy donors

The characteristics of the patients with CLL and the healthy donors are presented in **Table 1**.

**Table 1:** Characteristics of the patients and healthy donors.

Parameter	Primary patients with CLL	Healthy donors
Numbers, n	38	15
Age, years (median, range)	62 (44–83)	59 (39–82)
Stage according to Вinet*, n		
В	23	NA
С	10	NA

NA, not available. *Clinical data were available not to all 38 patients with CLL.

### Analysis of serum cytokines

**Figure 1:**
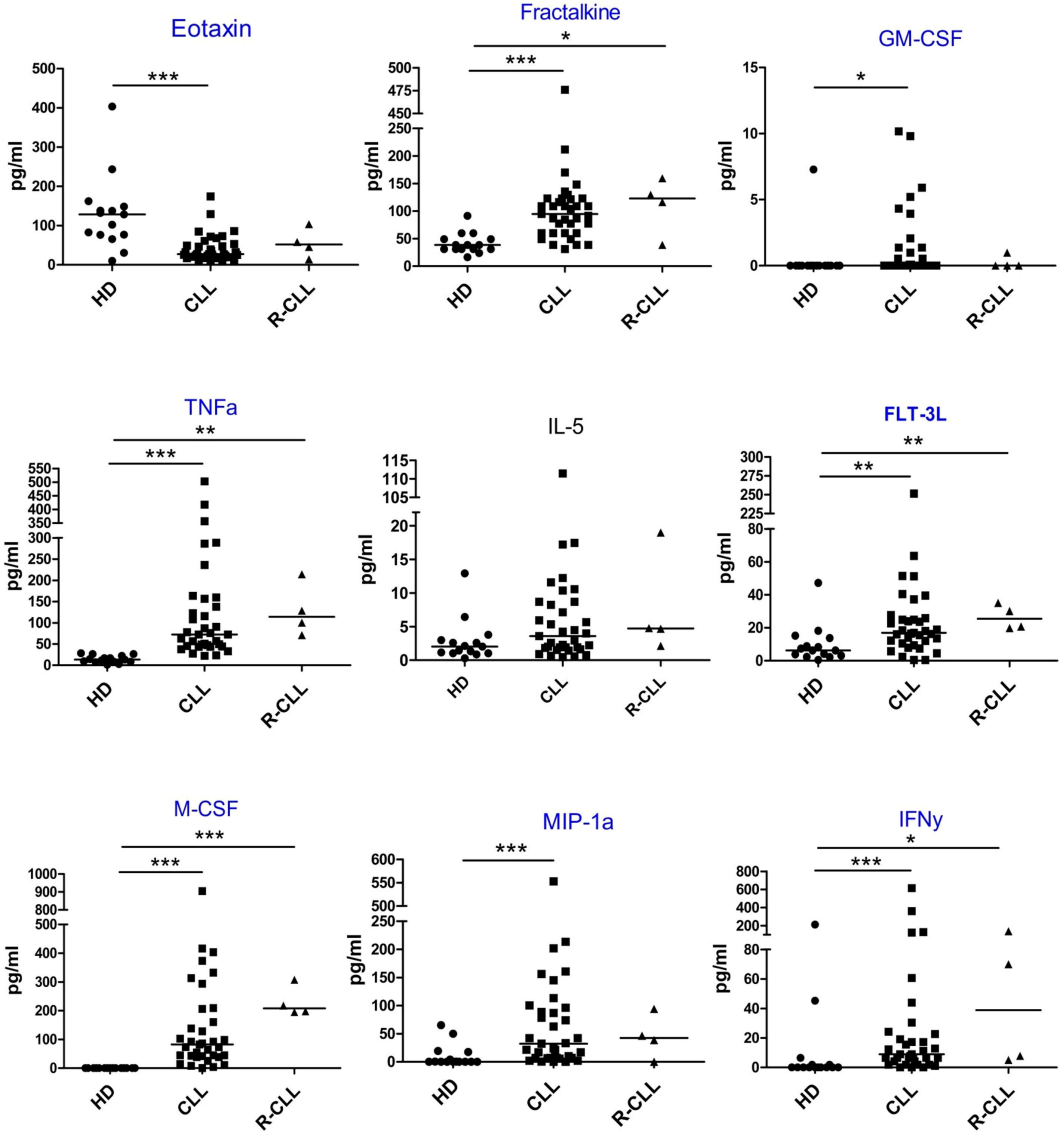
Serum cytokine levels in the study groups. HD – healthy donors (n=15), CLL – primary patients with CLL (n=35), R-CLL – patients with relapses CLL (n=4). The Kruskal-Wallis test was used for comparison among the three groups. P-values are as follows: Eotaxin, p=0.0031; Fractalkine, p=0.0007; GM-CSF, p=0.0596; TNFα, p<0.0001; IL-5, p=0.1078; FLT-3L, p=0.0012; M-CSF, p<0.0001; MIP-1α, p=0.0203; IFNγ, p=0.0125. Cytokines with significant differences between the primary CLL patients and healthy donors are highlighted. *p < 0.05; **p < 0.01; ***p < 0.001.

Serum levels of 47 cytokines were assessed in primary patients with CLL, patients with relapsed CLL, and healthy donors. The data are presented in **Figure 1** and the **Supplementary Materials**. Compared to healthy donors, patients with primary CLL exhibited significantly higher levels of FLT-3L, Fractalkine, G-CSF, GM-CSF, IFNα2, IFNγ, IL-2, IL-3, IL-4, IL-6, IL-9, IL-10, IL-12 (p40), IL-12 (p70), IL-15, IL-17E/IL-25, IL-18, IL-22, IL-27, IP-10, MCP-3, M-CSF, MDC, MIG, MIP-1α, MIP-1β, and TNFα. Conversely, the levels of sCD40L and Eotaxin were significantly lower in the primary CLL group compared to healthy donors.

### Flow cytometry

**Figure 2:**
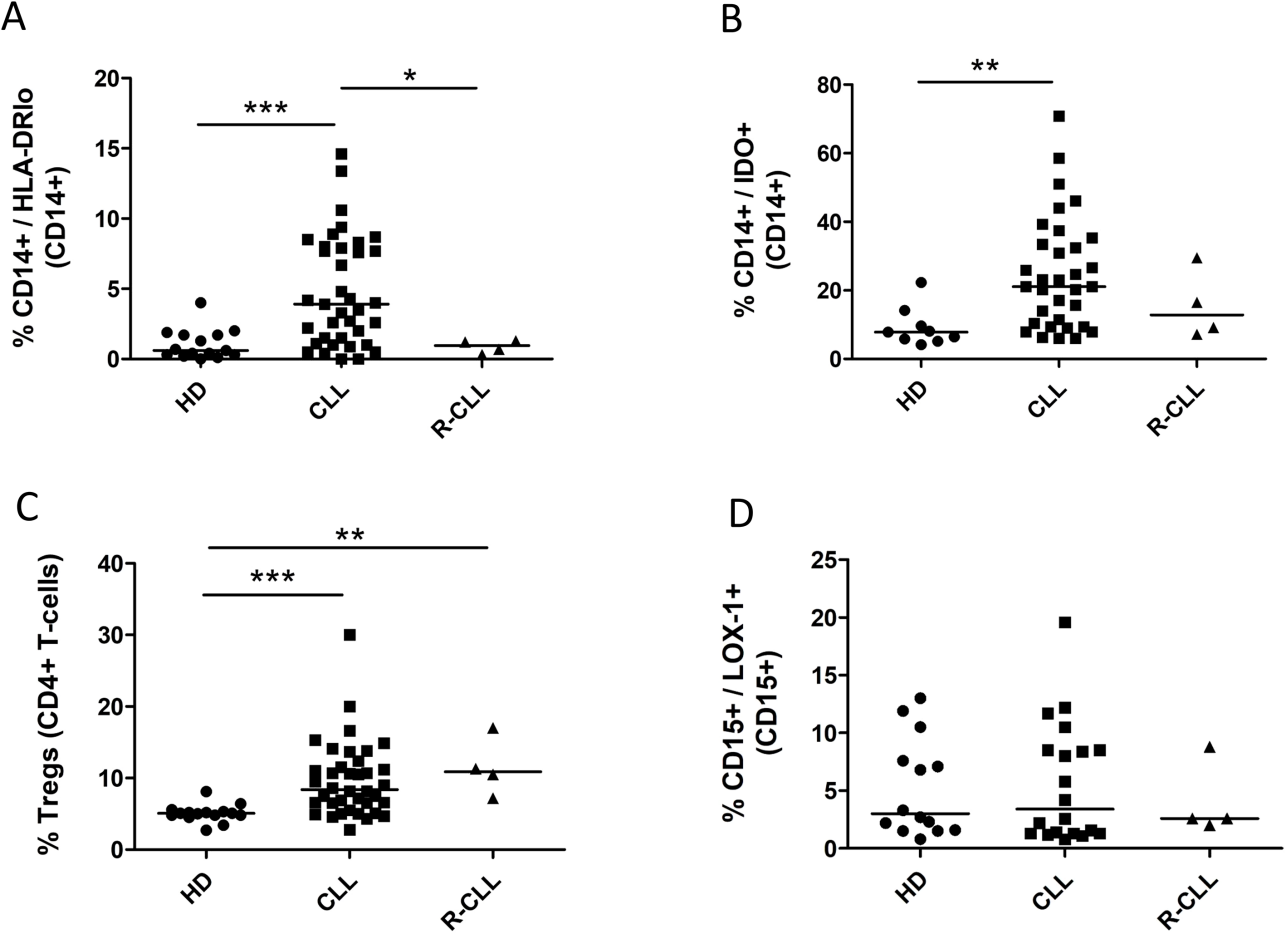
The frequencies of immunosuppressive cells in the peripheral blood of healthy donors (HD), primary patients with CLL (CLL) and patients with CLL relapses (R-CLL). **(A)** The frequencies of mMDSCs, with HD n=15, CLL n=37, R-CLL n=4. **(B)** The frequencies of CD14+/IDO+ cells, with HD n=9, CLL n=33, R-CLL n=4. **(C)** The frequencies of Tregs, with HD n=15, CLL n=38, R-CLL n=4. **(D)** The frequencies of gMDSCs, with HD n=14, CLL n=20, R-CLL n=4. The Kruskal-Wallis test results in three groups by: mMDSC p=0.0001, CD14+/IDO+ p=0.0117, Treg p=0.0010, gMDSC p=0.5258. *p < 0.05; **p < 0.01; ***p < 0.001.

**Figure 3:**
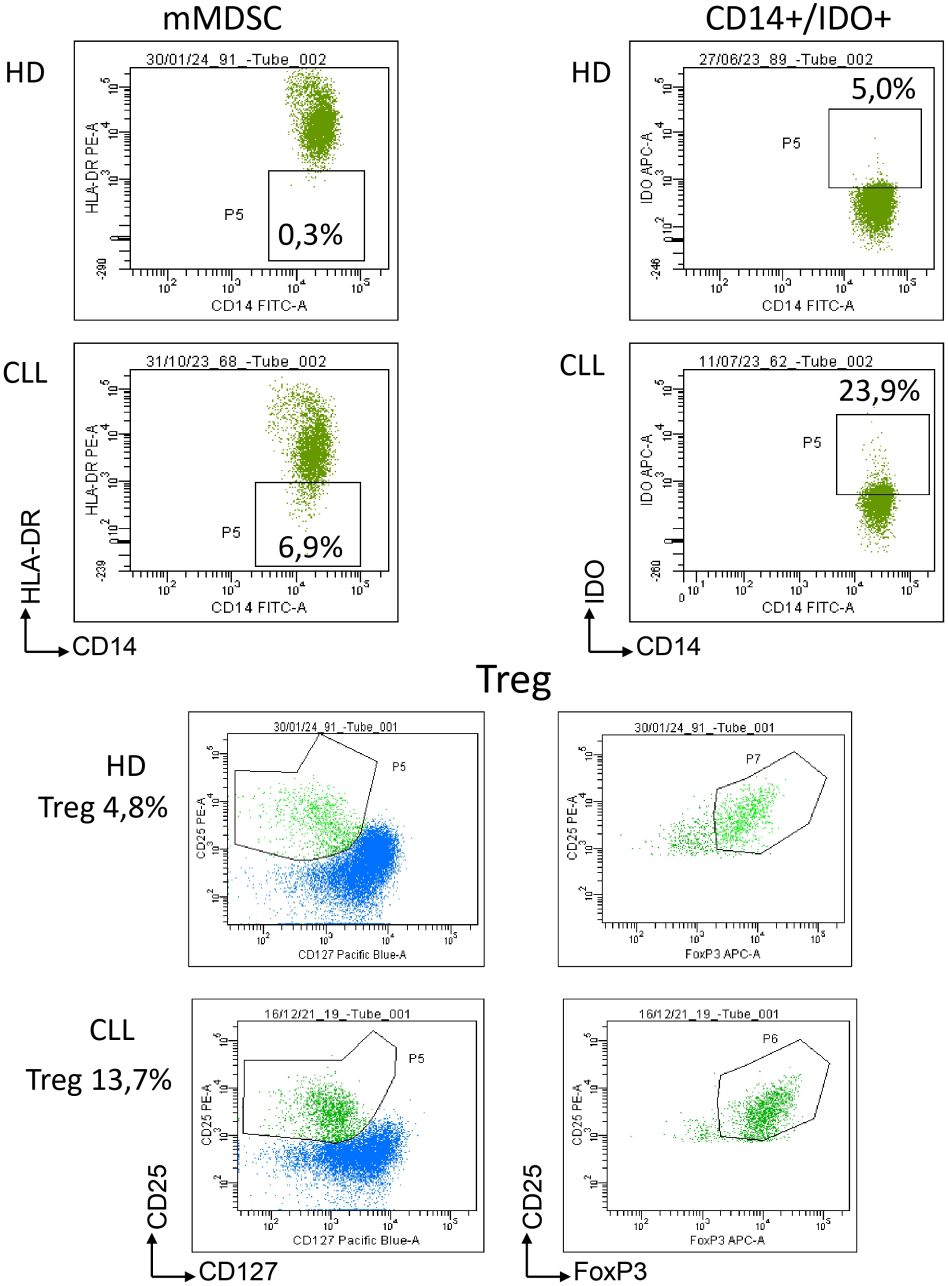
Representative DotPlots of mMDSCs, CD14+/IDO+cells and Tregs.

The graphs present the frequencies of immunosuppressive cells in the peripheral blood of healthy donors and patients with CLL (**Figure 2**). The frequencies of mMDSCs, CD14+/IDO+, and Treg cells were significantly higher in primary CLL patients than in healthy donors. The frequencies of gMDSCs did not significantly differ between primary patients and healthy donors. Only Tregs showed statistically significant differences between donors and patients with relapsed CLL. **Figure 3** presents Dot Plots of mMDSCs, CD14+/IDO+ cells, and Tregs in healthy donors and patients with CLL.

### Correlation

The Spearman correlation coefficient was used to determine the relationship between the numbers of immunosuppressive cells and serum cytokine levels in primary patients with CLL. Correction for multiple comparisons was performed using the false discovery rate (FDR). **Tables 2**–**5** present the results of the comparative analysis with p < 0.05 (N represents the number of pairs).

**Table 2:** Correlation of cytokines with mMDSC numbers.

Cells & cytokines	Valid, N	Spearman, R	P-value	P-value after FDR
mMDSC & GM-CSF	34	0.3635	0.0346	0.4061
mMDSC & M-CSF	34	0.3791	0.0270	0.4061

**Table 3:** Correlation of cytokine levels with the of CD14+/IDO+monocyte numbers.

Cells & cytokines	Valid, N	Spearman, R	P-value	P-value after FDR
CD14+/IDO+cells & IL-5	30	0.3925	0.0319	0.7497

**Table 4:** Correlation of cytokine levels with Treg numbers.

Cells & cytokines	Valid, N	Spearman, R	P-value	P-value after FDR
Treg & Eotaxin	35	-0.3569	0.0353	0.1106
Treg & FLT-3L	35	0.4436	0.0076	**0.0446**
Treg & Fractalkine	35	0.5356	0.0009	**0.0123**
Treg & IFNγ	35	0.3868	0.0217	0.0901
Treg & IL-10	35	0.4268	0.0106	0.0551
Treg & IL-12(p40)	35	0.3834	0.0230	0.0901
Treg & IL-12(p70)	35	0.3760	0.0260	0.0941
Treg & IL-18	35	0.4875	0.0030	**0.0233**
Treg & IL-27	35	0.4443	0.0075	**0.0446**
Treg & IP-10	35	0.3672	0.0300	0.1007
Treg & M-CSF	35	0.3888	0.0210	0.0901
Treg & MIG	35	0.5785	0.0003	**0.0064**
Treg & MIP-1α	35	0.5009	0.0022	**0.0205**
Treg & MIP-1β	35	0.5927	0.0002	**0.0064**
Treg & TNF-α	35	0.5302	0.0011	**0.0123**

Bold p-values correspond to significant statistical correlation after FDR correction.

**Table 5:** Correlation of cytokine levels with gMDSC numbers.

Cells & cytokines	Valid, N	Spearman, R	P-value	P-value after FDR
gMDSC & GROα	17	0.5396	0.0254	0.2719
gMDSC & IFNγ	17	0.6450	0.0052	0.2436
gMDSC & IL-1β	17	0.5377	0.0260	0.2719
gMDSC & IL-12(p40)	17	0.4954	0.0432	0.3382
gMDSC & IL-17A	17	0.5377	0.0260	0.2719
gMDSC & MIP-1α	17	0.5292	0.0289	0.2719

Although not all correlations were confirmed by FDR, we cannot claim that they are false (i.e., occasional). Such correlations should be noted as weak and analyzed cautiously.

Multivariate regression analysis revealed significant interrelations between 47 cytokines and immune cell subsets (**Table 6**). Stepwise regression revealed the most significant factors related to the number of mMDSCs, with an R² of 0.36. Increased numbers of mMDSCs were associated with higher levels of M-CSF and GM-CSF, while decreased mMDSC numbers were associated with a higher IL-10 level. The coefficient of determination for CD14+/IDO+monocytes was R² = 0.11. Higher CD14^+^/IDO^+^cell frequencies were associated with a higher IL-5 level. The analysis of the relationship between Tregs and the 47 cytokines showed an R² of 0.48. Increased Treg numbers correlated with higher MIP-1β and MIG levels, whereas decreased Treg frequencies correlated with higher eotaxin levels. The relationship between gMDSCs and the 47 cytokines showed an R² of 0.35. Increased gMDSC frequencies were associated with higher IFNγ levels.

**Table 6:** Regression model for cytokines and immunosuppressive cells.

Dependent variable	Independent variables	b	SE b	Beta	t	P-value	VIF
mMDSC	(Constant)	11.94	4.01		2.98	0.006	
	M-CSF	0.52	0.17	0.49	3.10	0.004	1.29
	IL-10	-0.54	0.17	-0.51	-3.24	0.003	1.28
	GM-CSF	0.42	0.18	0.36	2.37	0.024	1.16
CD14+/IDO+	(Constant)	11.06	3.50		3.16	0.004	
	IL-5	0.36	0.17	0.38	2.14	0.041	1.00
Treg	(Constant)	11.05	4.20		2.63	0.013	
	MIP-1β	0.38	0.16	0.36	2.46	0.020	1.36
	MIG	0.40	0.15	0.37	2.58	0.015	1.33
	Eotaxin	-0.30	0.14	-0.28	-2.21	0.034	1.03
gMDSC	(Constant)	4.00	1.92		2.08	0.055	
	IFNγ	0.30	0.10	0.62	3.09	0.007	1.00

### Cytokines at different stages of CLL

**Table 7** presents those cytokines from the panel of 47 studied that showed statistically significant differences in their levels between primary patients with CLL at stages B and C according to the Binet classification. With the exception of PDGF-AB/BB, all cytokines exhibited higher levels in patients at stage C compared with those at stage B.

**Table 7:** Cytokine levels in primary patients with CLL at stages B and C.

Cytokines	В pg/mL (n=23), median (Q1-Q3)	С pg/mL (n=10), median (Q1-Q3)	p-value
FGF-2	38 (22–84)	318 (104-1497)	0.0017
G-CSF	25 (19-43)	68 (29-165)	0.0376
IL-1β	0 (0-0)	9 (0-154)	0.0383
IL-9	10 (8-15)	17 (9-39)	0.0436
IL-10	6 (1-15)	21 (10-30)	0.0076
IL-12(p70)	1 (0-2)	4 (1-16)	0.0278
IL-15	13 (9-16)	17 (13-34)	0.0282
IL-17A	0 (0-0)	0 (0-39)	0.0015
IL-18	76 (43-170)	206 (154-709)	0.0097
IL-27	7135 (5556-9287)	9724 (8138-17185)	0.0109
M-CSF	64 (25-128)	250 (87-407)	0.0054
MIP-1α	22 (7-79)	98 (15-171)	0.0434
PDGF-AB/BB	46759 (28506-134265)	27153 (18526-33021)	0.0136

## Discussion

In the present study, the levels of 47 cytokines were measured in patients with CLL and healthy donors. Of these, 29 cytokines showed significantly different levels between primary patients and healthy donors (**Figure 1** and **Supplementary Materials**). Yan XJ et al. evaluated the levels of 23 cytokines and also noted that more than half of them exhibited significant differences between patients with CLL and healthy donors (17). However, data in the literature may be discrepant regarding individual cytokines. For instance, the present study found no statistically significant difference in IL-1β levels between donors and primary patients (**Supplementary Materials**). Gonzalo-Blanco et al. also reported that IL-1β levels do not differ between donors and patients (18). In contrast, one study reported significantly higher IL-1β levels in patients with CLL compared with normal controls (17), while another study showed that plasma IL-1β concentrations were significantly lower in patients with CLL than in healthy donors (20). These discrepancies may be attributable to differences in the proportion of patients at a particular disease stage. The present study revealed statistically significant differences in IL-1β levels between patients at stages B and C of the Binet classification (**Table 6**). Previous studies have shown that cytokines can support the viability of CLL cells *in vitro*, e.g., IFNγ (21), which was elevated in our studies, or MCP-1 (CCL2) (22), which was not increased in our experiments.

Although we assessed and presented data on immunosuppressive cells and cytokines in patients with relapsed CLL, the findings are only of research interest due to the small sample size (n=4) and, therefore, will not be discussed further in this section.

We found a statistically significant increase in the numbers of mMDSCs and Tregs in primary patients with CLL compared with healthy donors (**Figure 2**). These findings are consistent with those of previous studies (8, 14). Jitschin et al. analyzed a small cohort and demonstrated higher expression of IDO in patient monocytes than in donor monocytes (8). The present study confirmed these findings in a larger cohort and demonstrated that the number of CD14+/IDO+monocytes was significantly higher in primary patients with CLL than in healthy donors (**Figure 2**). Several studies have reported an increased number of CD15+/LOX-1+gMDSCs in the peripheral blood of patients with solid tumors (23, 24). However, the present study found no differences in the number of these cells between patients with CLL and healthy donors (**Figure 2**). In contrast, Ferrer et al. noted an increase in the number of gMDSCs with an HLA-DRlo/CD11b+/CD33+/CD14-/CD15+ phenotype in patients with CLL (9). It is possible that patients with CLL exhibit a different gMDSC phenotype than do patients with solid tumors. According to the literature, increased numbers of mMDSCs (13, 14), HLA-DRlo/CD11b+/CD33+/CD14-/CD15+ gMDSCs (9) and Tregs (15, 16) are associated with poor prognosis in CLL. Furthermore, Öztürk S. et al. showed that IDO1 targeting therapy does not control CLL in a mouse model (25).

The present study evaluated the relationship between the numbers of immunosuppressive cells and levels of cytokines in patients with CLL using Spearman’s correlation coefficient. Some correlations were not confirmed by the FDR adjustment; however, this does not necessarily imply that all of them are spurious. Rather, it suggests that these correlations are weak and should be interpreted with caution. Indeed, some authors argue that it is not always reasonable to consider only data that remain significant after adjustment for multiple comparisons (26). Besides, some authors consider that investigational research may not require strict multiple-testing adjustments (27). Since the presented investigational study yielded weak correlations, it would be worthwhile to replicate these findings in a larger sample. Nevertheless, a number of these weak correlations were confirmed by the multiple regression analysis in the present study. Moreover, literature data support biological relationships between several of the cytokines and immunosuppressive cells that exhibited weak correlations.

The present study found that the number of mMDSCs positively correlated with the levels of GM-CSF and M-CSF, though after FDR adjustment these correlations lost statistical significance (**Table 2**). On the other hand, they were confirmed by regression analysis (**Table 6**). Moreover, some publications reported biological relationship between mMDSC and GM-CSF. For instance, administration of a GM-CSF vaccine to cancer patients led to an increase in mMDSC numbers (28). Other studies have shown that culturing peripheral blood monocytes *in vitro* with IL-6 and GM-CSF promotes the generation of immunosuppressive mMDSCs (29, 30).

The correlations, which we observed before FDR adjustment, were consistent with the hypothesis that the addition of growth factors to secreted pro-inflammatory cytokines promotes immunosuppression and, consequently, the accumulation of mMDSCs (4). Furthermore, the present study demonstrated elevated levels of pro-inflammatory cytokines (**Figure 1** and **Supplementary Materials**).

Notably, regression analysis also revealed an inverse correlation between mMDSCs and IL-10 (**Table 6**), a finding that seems unusual at first glance. IL-10 is known to be an anti-inflammatory and immunosuppressive cytokine. According to the literature, inflammation in oncology is associated with immunosuppression (2, 3), and strategies aimed at neutralizing inflammation may have a therapeutic effect (31). It is possible that elevated IL-10 leads to a decrease in inflammation and thus indirectly contributes to a reduction in mMDSCs. At the same time, we found a positive correlation between IL-10 and Tregs (**Table 4**), which is consistent with the immunosuppressive functions of IL-10. This suggests that, in this context, IL-10 directly affects Tregs. An experimental study has shown that IL-10 suppression enhanced antitumor immunity in a CLL model (32). These data are more consistent with the data on the paired positive correlation of IL-10 and Tregs.

We found that the numbers of CD14+/IDO+monocytes positively correlated with IL-5, however, this correlation lost statistical significance after FDR adjustment (**Table 3**). Nevertheless, the revealed correlation was confirmed by regression analysis (**Table 6**).

The study showed that Tregs negatively correlated with Eotaxin and positively correlated with FLT-3L, Fractalkine, IFNγ, IL-10, IL-12(p40), IL-12(p70), IL-18, IL-27, IP-10, M-CSF, MIG, MIP-1α, MIP-1β, TNFα. After FDR adjustment, statistically significant correlations are considered those between Tregs and FLT-3L, Fractalkine, IL-18, IL-27, MIG, MIP-1α, MIP-1β, TNFα (**Table 4**). Notably, regression analysis confirmed the correlation between Tregs and such cytokines as Eotaxin, MIP-1β, and MIG (**Table 6**). Literature data demonstrated biological relationship between Tregs and cytokines similar to those found in our study both before and after FDR adjustment. The literature indicated that administration of FLT-3L to cancer patients led to the increase in numbers of CD4+ FoxP3+Tregs in the peripheral blood (33). These data support our results on the positive correlation of FLT-3L and Tregs. At the same time, in case of patients with excessive immune activation, such as systemic lupus erythematosus (SLE), the data showed an inverse correlation of Tregs and Fractalkine. In patients with SLE, the amount of Tregs in the serum decreases, and the level of Fractalkine increases (34). That observation was different from our data from patients with CLL, where we registered a positive correlation between the Treg frequencies and Fractalkine levels. However, this discrepancy can be resolved by considering the underlying hypothesis. In the context of excessive immune system activation, pro-inflammatory cytokines are found at predominant concentrations, whereas growth factors are present only in small amounts. If this condition corresponds to that observed in SLE, then an increase in the level of the pro-inflammatory cytokine Fractalkine would be expected to weaken immunosuppression (i.e., reduce the number of Tregs). Conversely, in patients with CLL—where immunosuppression occurs alongside a simultaneous increase in both growth factors and pro-inflammatory cytokines—an increase in Fractalkine would be expected to enhance immunosuppression (i.e., increase the number of Tregs).

Regarding IL-12(p70), it has been reported to promote the induction of CD4+CD25+ Tregs that inhibit allograft rejection in animal models (35). Oertli et al. found that IL-18 played an important role in T-cell differentiation into Tregs in a mouse model of H. pylori infection (36). Lunardi et al. reported that *IP-10* expression in human pancreatic ductal adenocarcinoma (PDAC) was associated with intratumoral Tregs (37). Zhang et al. found that MIP-1α (CCL3) attracted Tregs to stimulate the repair of cryodamaged muscles in mice (38). Wang et al. conducted *in vitro* studies and showed that TNFα increased the number of Tregs in patients with acute myeloid leukemia (AML) and in healthy donors (39). All of the above findings are consistent with our data showing a positive correlation between the levels of these cytokines and Treg numbers.

However, the pleiotropic nature of various cytokines should also be noted, as it may explain the conflicting results obtained under different conditions. For example, in a mouse model of obesity, IL-18 directly inhibited Treg suppressive function by reducing FoxP3 expression (40).

The observed negative correlation between Treg numbers and Eotaxin levels is particularly noteworthy, before FDR adjustment (**Table 4**). Moreover, we found that Eotaxin levels in treatment-naïve patients with CLL were significantly lower than those in healthy donors (**Figure 1**). This raises the question of whether peripheral blood eotaxin levels directly influence Treg numbers or whether this represents a spurious correlation. If a causal relationship is established, a therapeutic approach could involve administering Eotaxin to restore its normal concentrations, thereby potentially reducing Treg numbers.

The present study showed that gMDSCs positively correlated with the levels of GROα, IFNγ, IL-1β, IL-12(p40), IL-17A, and MIP-1α, however after FDR adjustment they appeared to be statistically insignificant (**Table 5**). Regression analysis confirmed correlation between gMDSCs and IFNγ only (**Table 6**). However, relationship between gMDSC and certain studied cytokines may be found in literature data. GROα is known to attract neutrophils to inflammatory sites. Combadière et al. found that immature LOX-1+ neutrophils were associated with the cytokine storm (involving IL-1β, IL-6, IL-8, and TNFα) in patients with COVID-19 (41). Based on our own findings before FDR and data from the literature, we assume that GROα and other pro-inflammatory cytokines play an important role in the accumulation of LOX-1+gMDSCs.

We found that the levels of such cytokines as FGF-2, G-CSF, IL-1β, IL-9, IL-10, IL-12 (p70), IL-15, IL-17A, IL-18, IL-27, M-CSF, MIP-1α, PDGF-AB/BB significantly differed between primary patients with CLL at stages B or C (**Table 7**). A higher stage indicates a more advanced disease and a higher risk of complications.

In summary, several cytokines of particular importance in CLL can be identified. Sivina et al. reported that plasma MIP-1α (CCL3) concentration is a reliable and independent prognostic marker for CLL (42). Other researchers have identified MIP-1α as the cytokine that best distinguishes CLL patients from healthy donors (17). Consistent with these findings, MIP-1α levels in the present study also differed significantly between patients with CLL and healthy donors (**Figure 1**). In addition, we found that MIP-1α levels in patients positively correlated with Tregs even after FDR adjustment (**Table 4**) and gMDSC before FDR (**Table 5**) numbers. Furthermore, we showed that MIP-1α levels differed significantly between patients at stages B and C of the Binet classification (**Table 6**). Thus, both the literature and our own results demonstrate the clinical importance of MIP-1α levels in patients with CLL.

In contrast, scarce information is available in the literature regarding the role of M-CSF in patients with CLL. The present study showed that patients and healthy donors had markedly different M-CSF levels; in fact, M-CSF was undetectable in healthy donors (**Figure 1**). Moreover, we found that M-CSF levels in patients positively correlated with mMDSC (**Table 3**) and Treg (**Table 4**) numbers before FDR adjustment. Relationship between mMDSCs and M-CSF was confirmed by regression analysis (**Table 6**). M-CSF levels also differed significantly between patients at stages B and C of the Binet classification (**Table 6**). These findings suggest that M-CSF is a promising candidate for further investigation as a prognostic marker in patients with CLL.

## Conclusion

In this study, we measured the levels of 47 cytokines in the serum of patients with CLL and healthy donors. Approximately half of these cytokines exhibited elevated levels compared to those in healthy donors. Our data confirmed that the numbers of mMDSCs, Tregs, and CD14+/IDO+ monocytes were increased in the peripheral blood of CLL patients. The number of gMDSCs in these patients did not differ significantly from that in healthy donors. We identified correlations between cytokine levels and the numbers of suppressor cells in CLL patients.

These findings provide valuable information for predicting the effects of therapeutic use or inhibition of the studied cytokines on suppressor cells in cancer patients, particularly given that the revealed relationship between GM-CSF and mMDSCs, as well as between FLT3L and Tregs, are entirely consistent with the literature. We also noted an important role for MIP-1α in CLL, which is in agreement with published data. Furthermore, we suggest that M-CSF may significantly influence immune suppression in CLL and warrants consideration as a prognostic marker in future studies.

This exploratory study revealed no substantial contradictions with the tested hypothesis implying that pro-inflammatory cytokines and growth factors acting together lead to immunosuppressive inflammation. Further studies could contribute to the understanding of the mechanism of the immunosuppressive inflammation.

## Data Availability

The original contributions presented in the study are included in the article/**Supplementary Material**. Further inquiries can be directed to the corresponding author.
