# Supplementary material for: Correlation between immune suppressor cells and cytokines in the peripheral blood of patients with chronic lymphocytic leukemia
Source: Front Oncol. 2026 Apr 15;16:1777390. doi: 10.3389/fonc.2026.1777390 (PMC13124494; doi:10.3389/fonc.2026.1777390)

Table S1. Monoclonal antibodies and fluorochromes used in the research

| Monoclonal antibody | Fluorochrome | Clone | Manufacturer |
| --- | --- | --- | --- |
| IDO | APC | eyedio | eBioscience |
| FoxP3 | APC | PCH101 | eBioscience |
| CD3 | PerCP | SK7 | BD Biosciences |
| CD4 | FITC | RPA-T4 | BD Biosciences |
| CD25 | PE | M-A251 | BD Biosciences |
| CD127 | BV421 | HIL-7R-M21 | BD Biosciences |
| CD14 | FITC | RMO52 | Beckman Coulter |
| HLA-DR | PE | L243 | BD Biosciences |
| CD45 | PE | HI30 | Sony |
| CD15 | eFluor450 | HI98 | eBioscience |
| LOX-1 | APC | 15C4 | Biolegend |

Table S2. Phenotypes of the studied populations of immunosuppressive cells

| Cell populations | Immunophenotype |
| --- | --- |
| mMDSC | CD14+/HLA-DRlow |
| IDO+monocytes | CD14+/IDO+ |
| Трег | CD3+/CD4+/CD25+/CD127−/FoxP3+ |
| gMDSC | CD45+/CD15+/ CD14−/LOX-1+ |


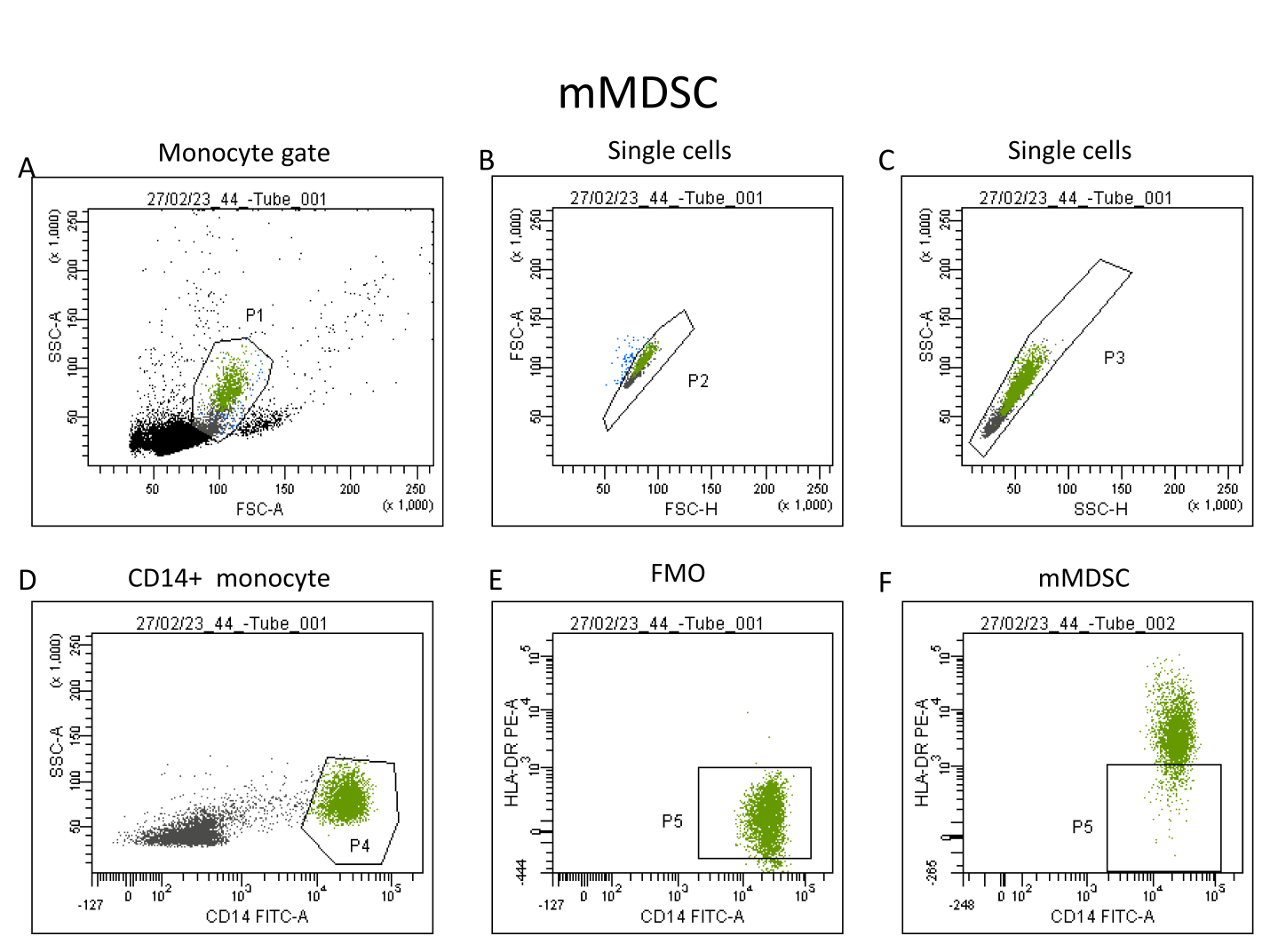


Figure S1. Gating strategy for mMDSC.


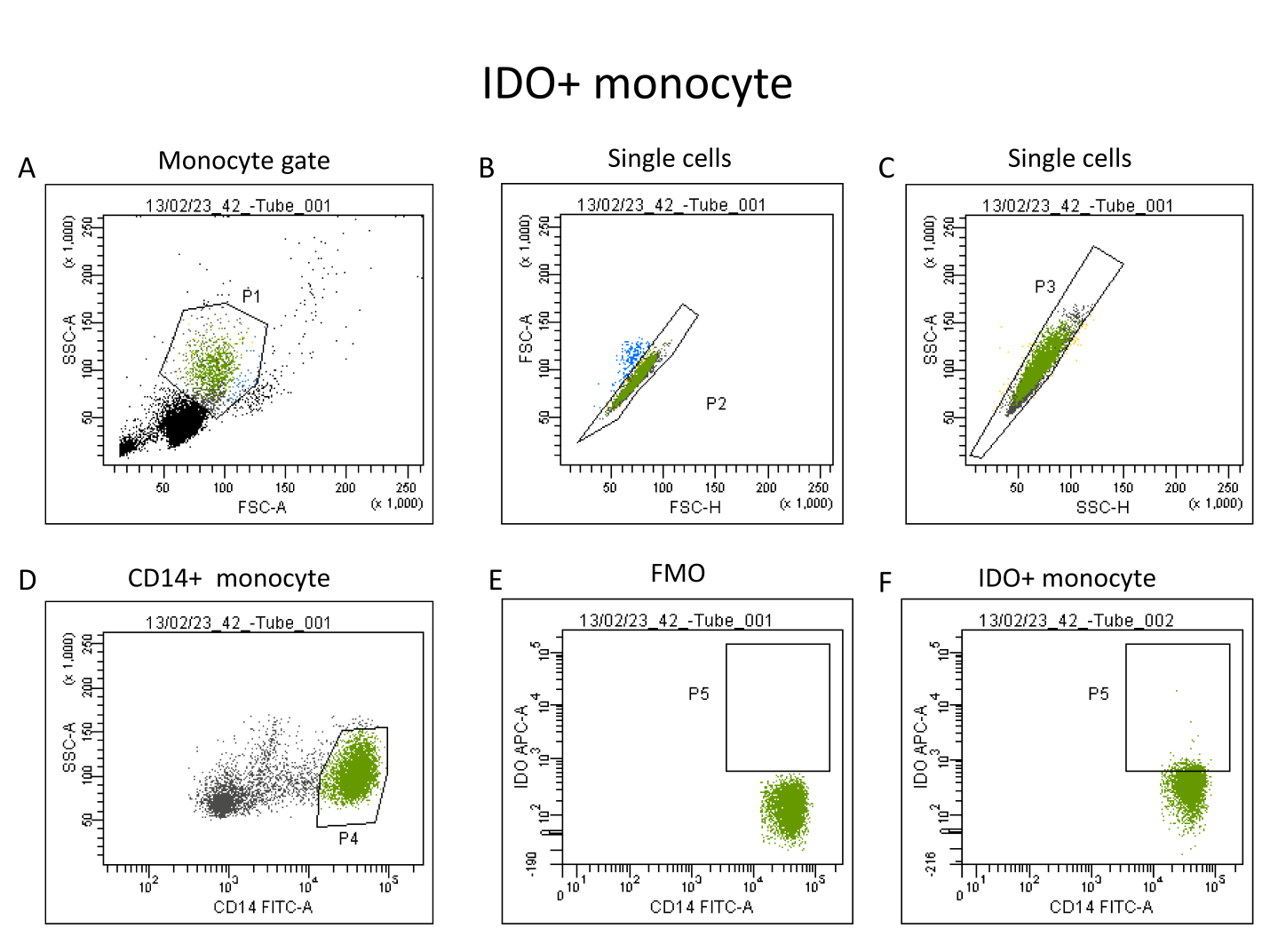


Figure S2. Gating strategy for IDO+monocytes


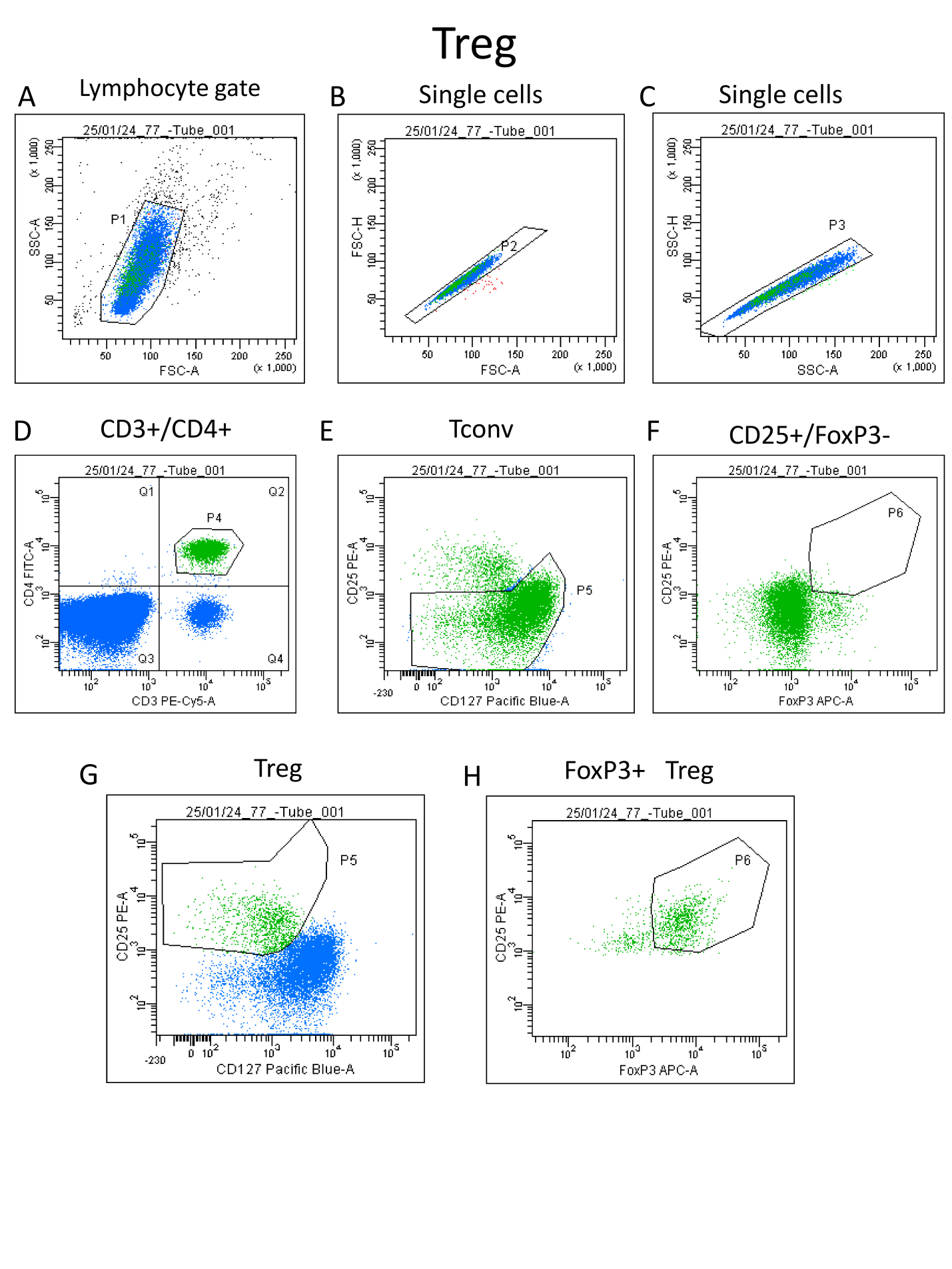


Figure S3. Gating strategy for Tregs


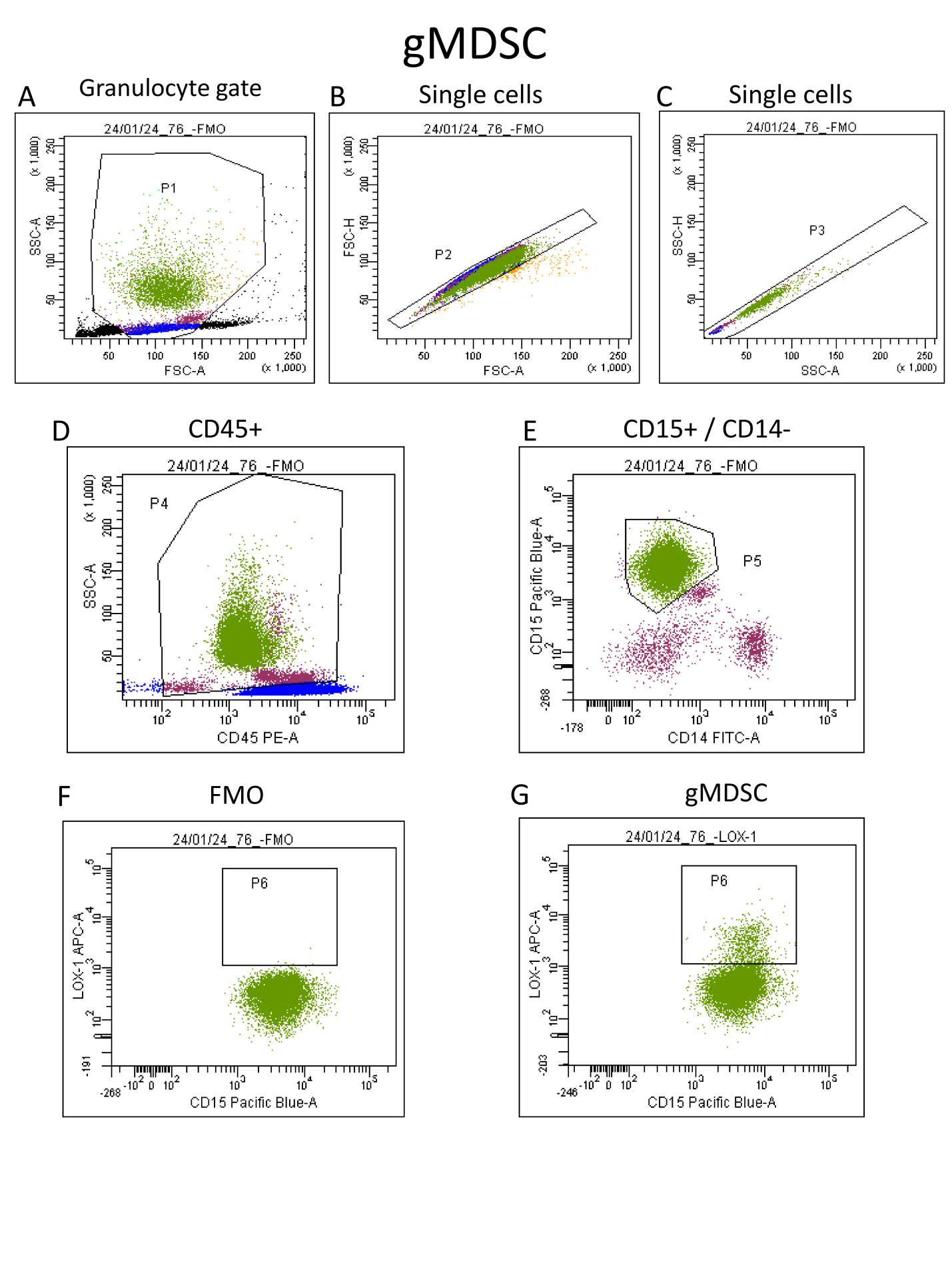


Figure S4. Gating strategy for gMDSCs

The data regarding the levels of serum cytokines are presented below.

Note: HD – healthy donors (n=15), CLL – primary patients with CLL (n=35), R-CLL - patients with the CLL relapse (n=4). Cytokine names are highlighted in color if there were statistically significant differences between the group of donors and primary patients with CLL. *p < 0.5; **p < 0.01; ***p < 0.001.


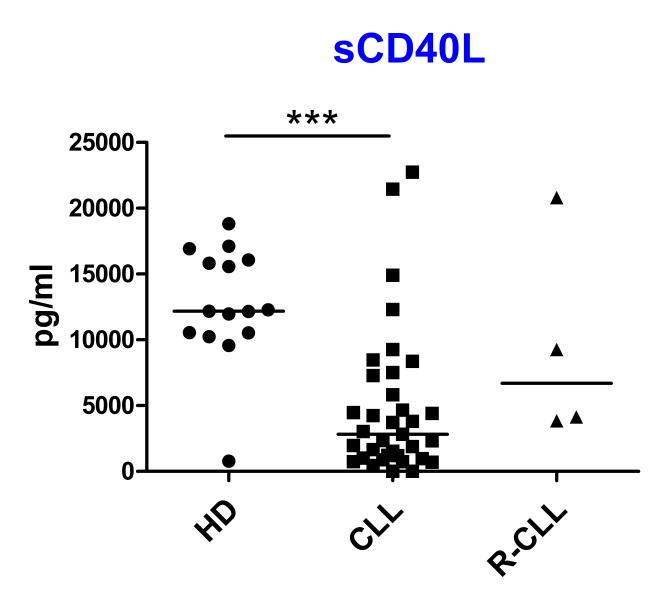

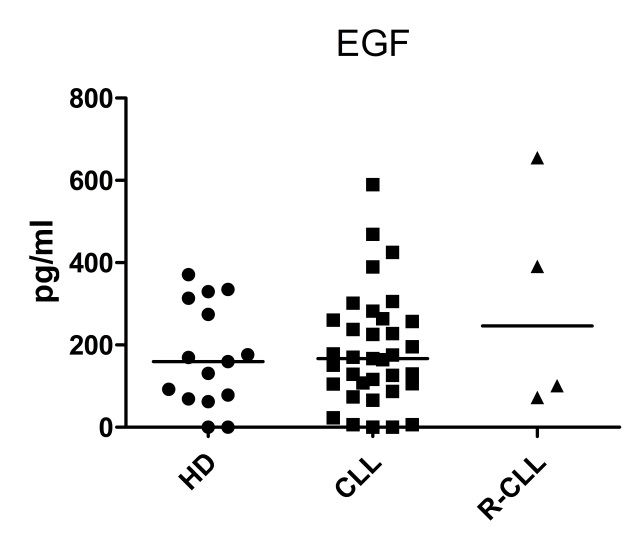


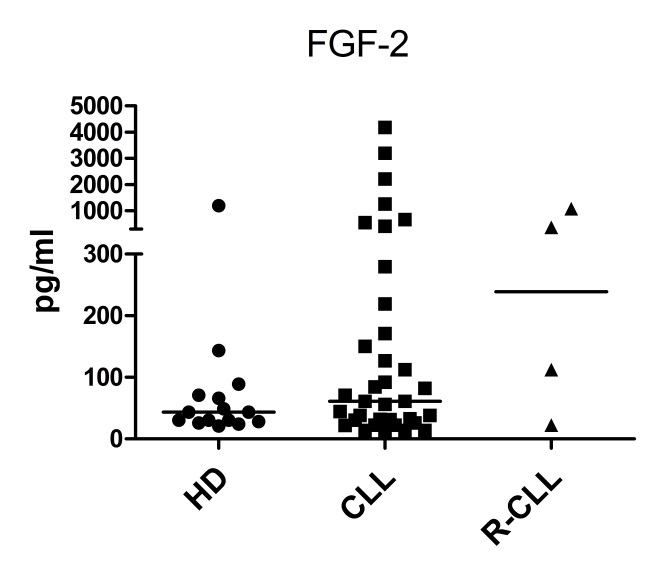

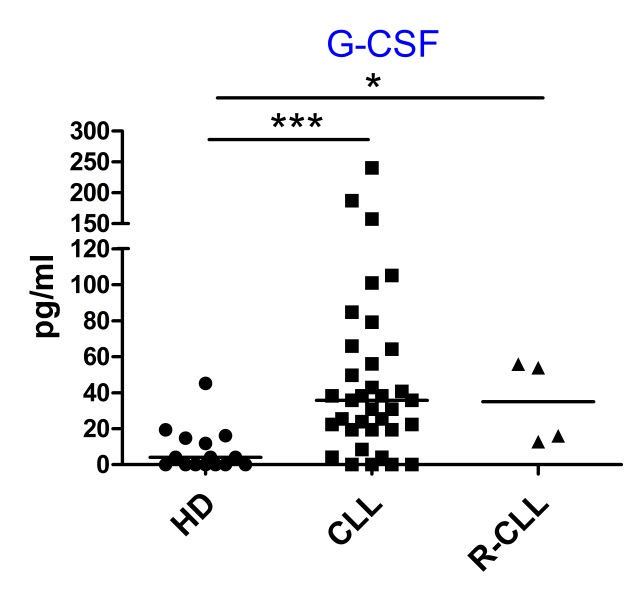


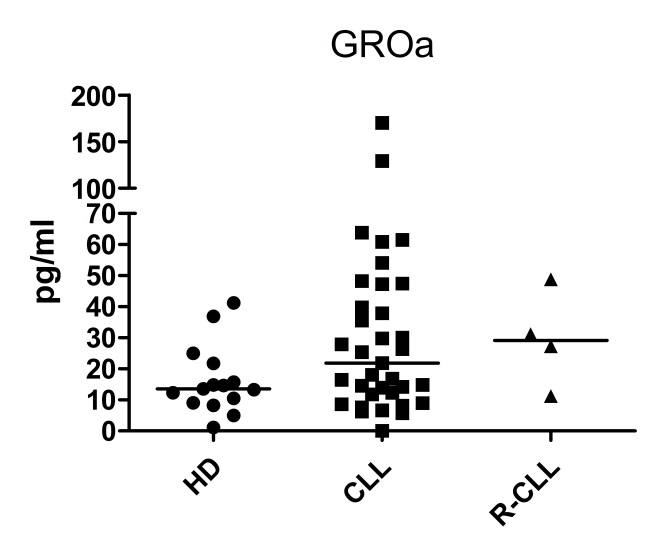

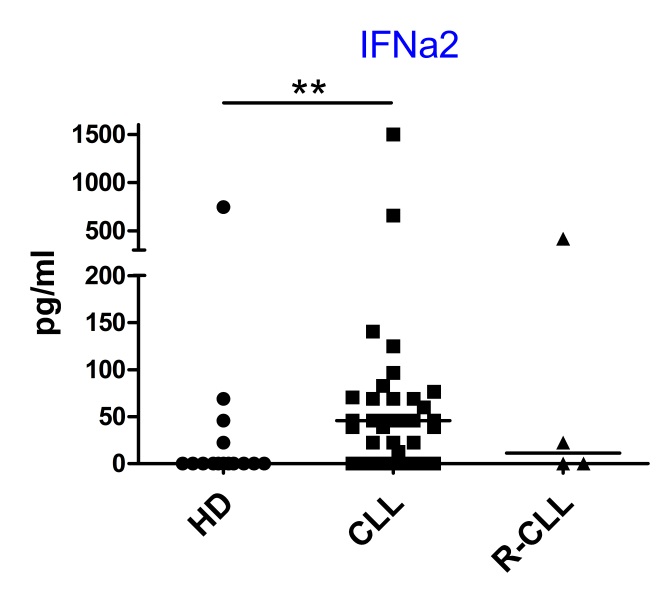


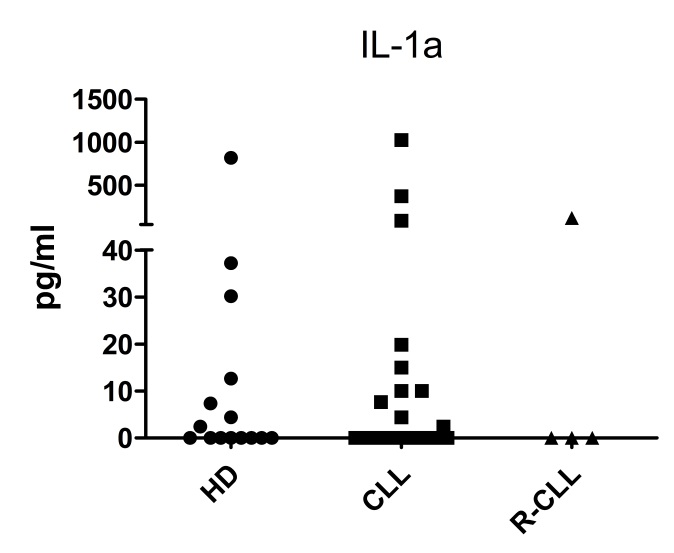

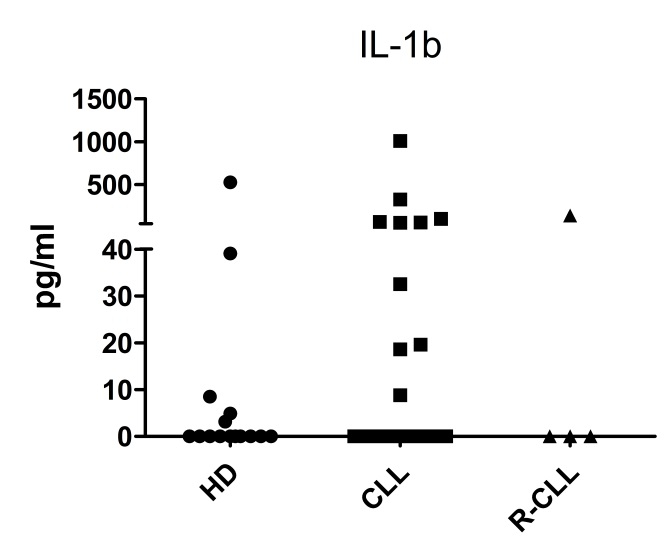


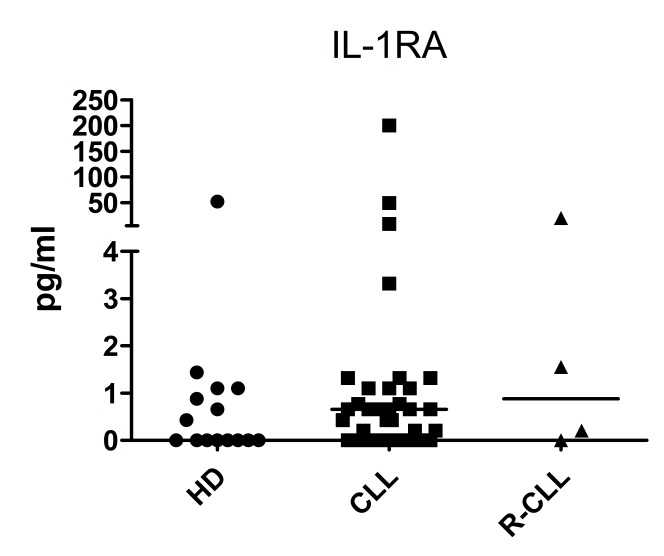

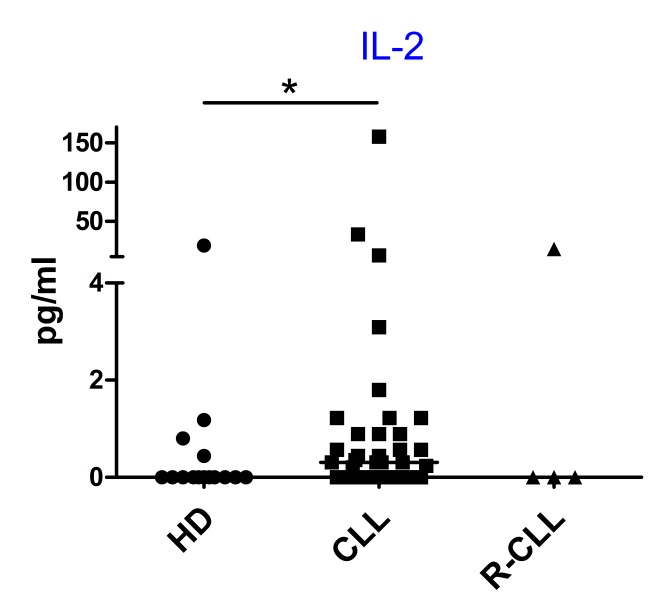


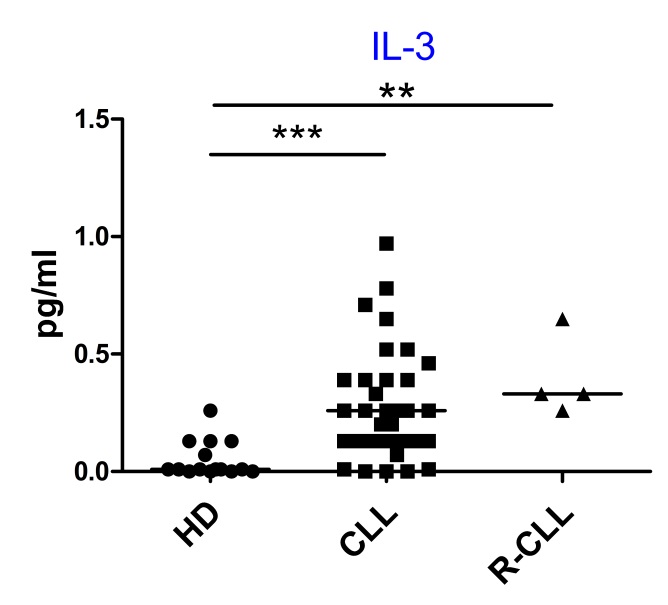

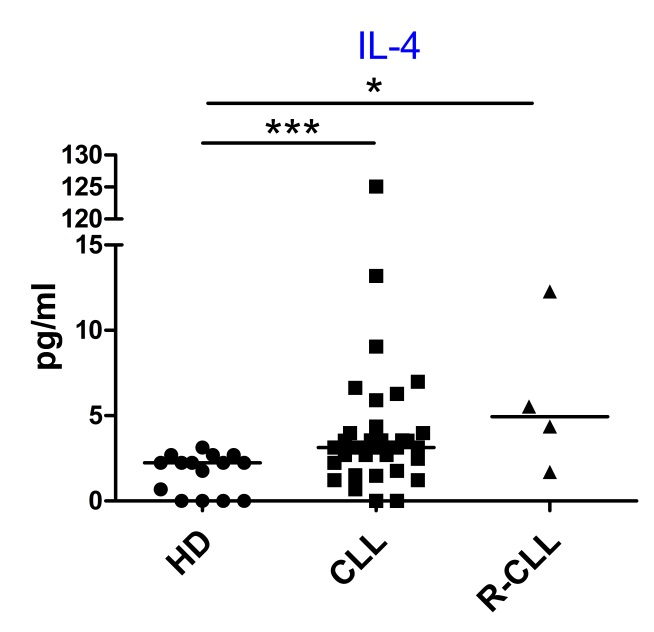


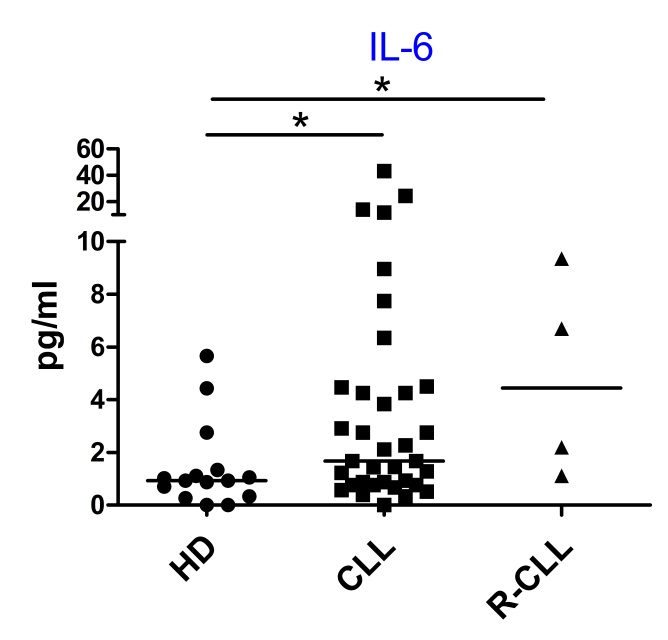

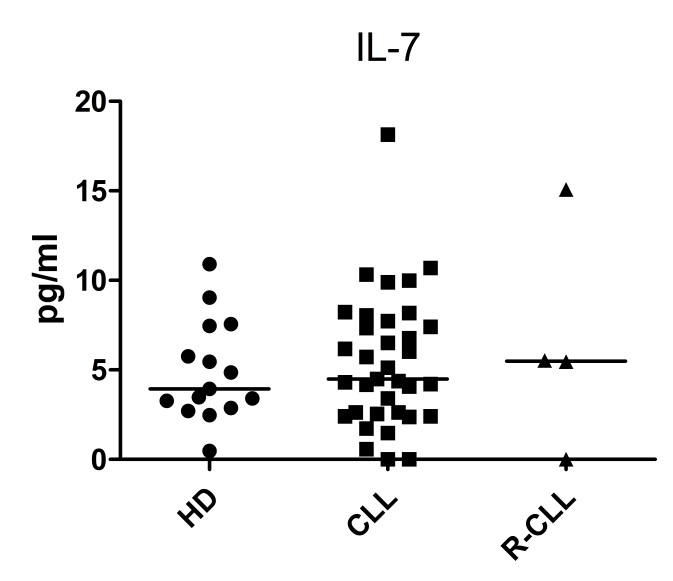


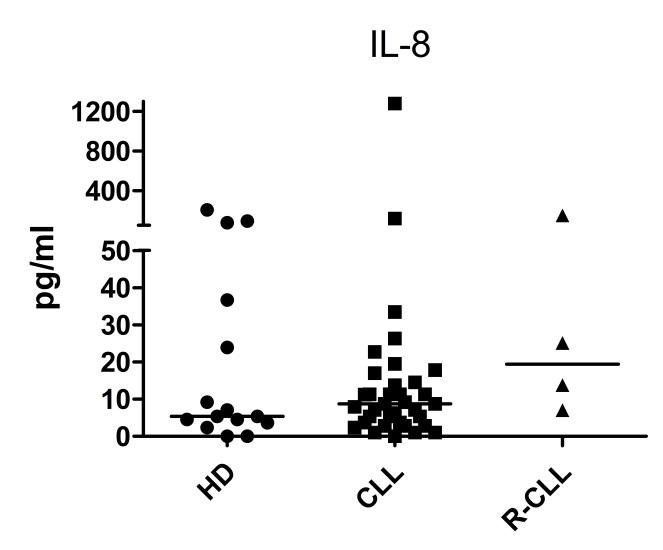

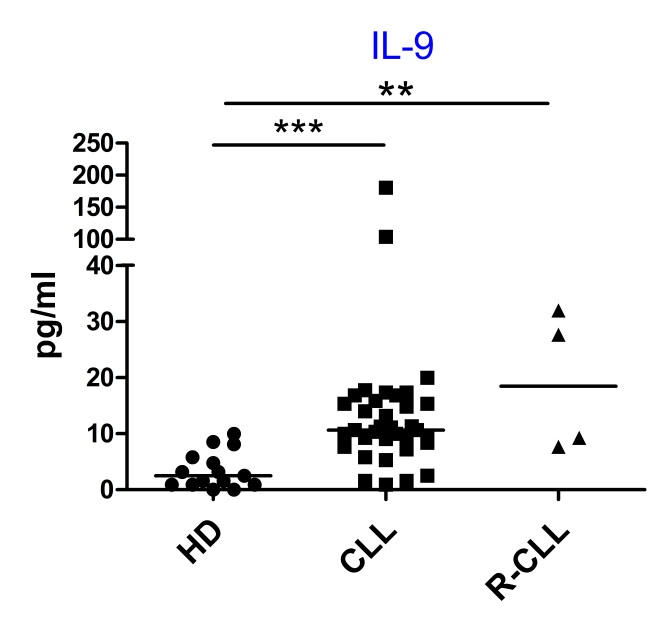


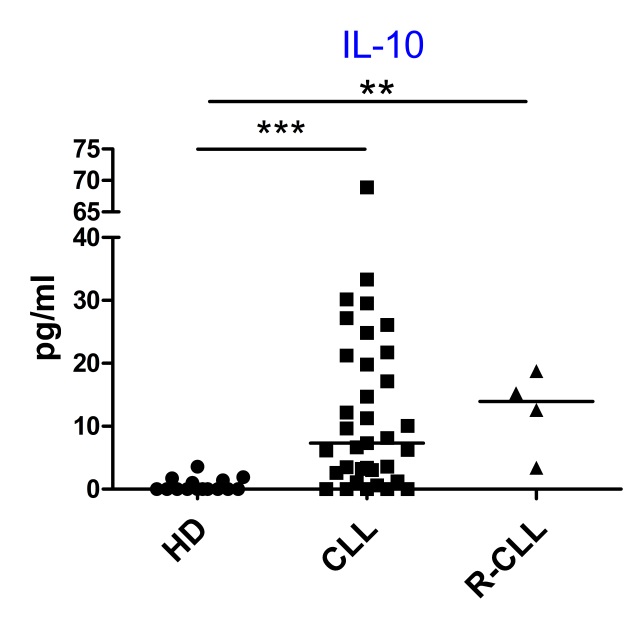

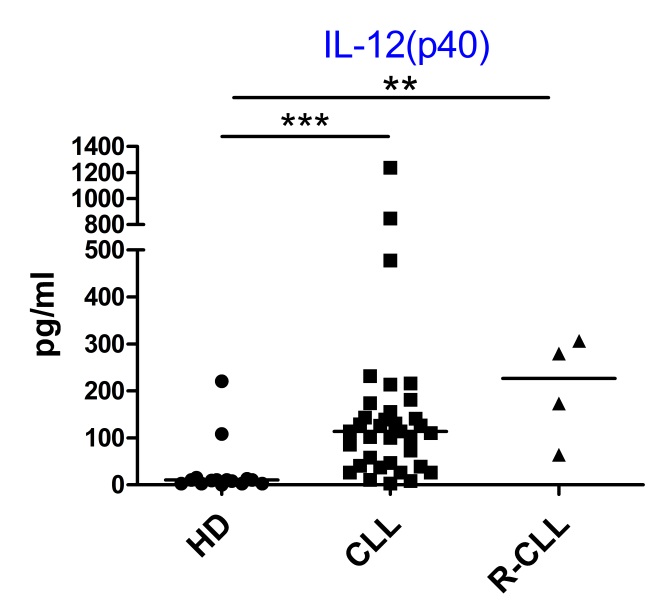


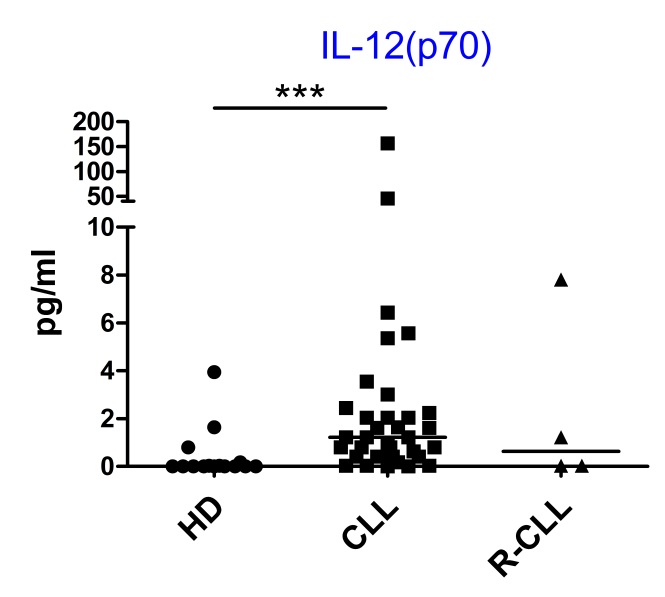

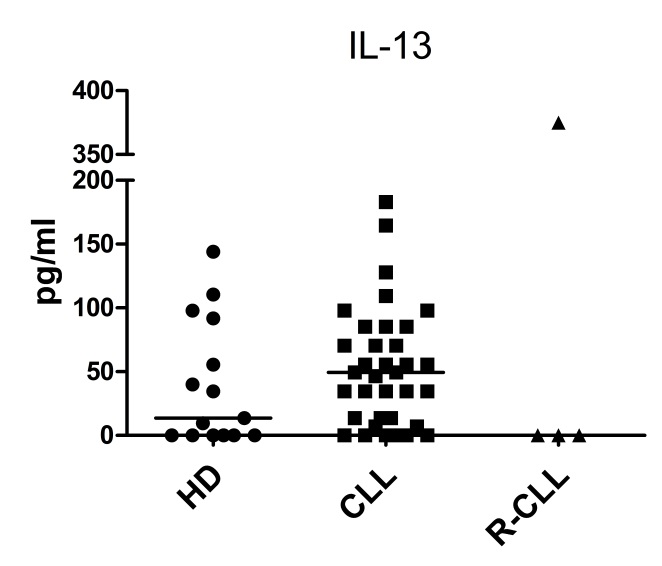


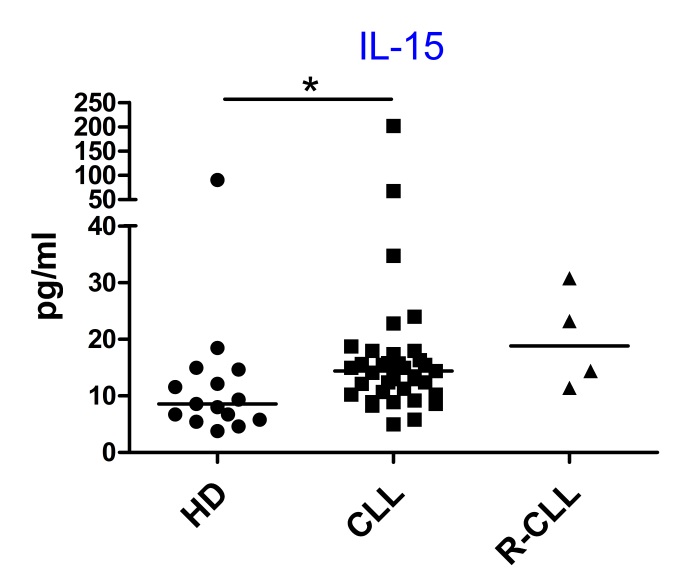

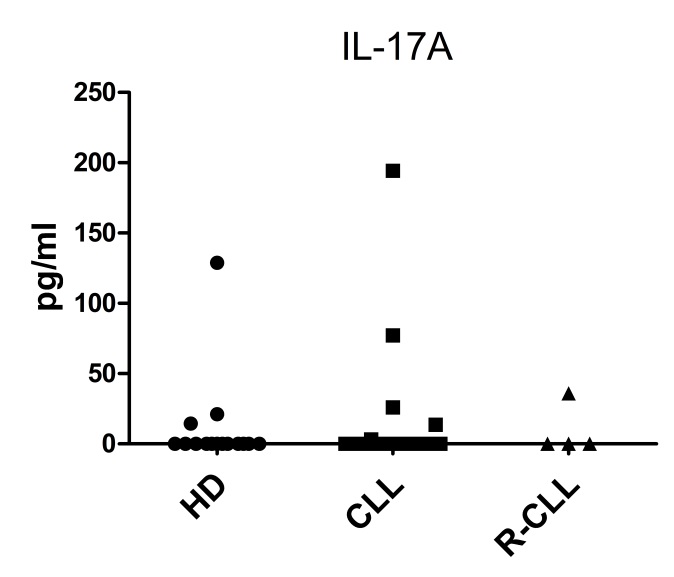


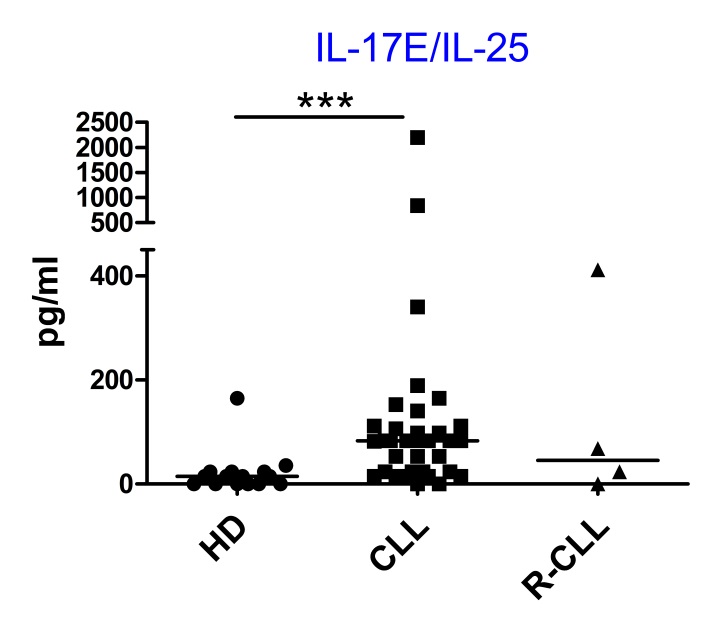

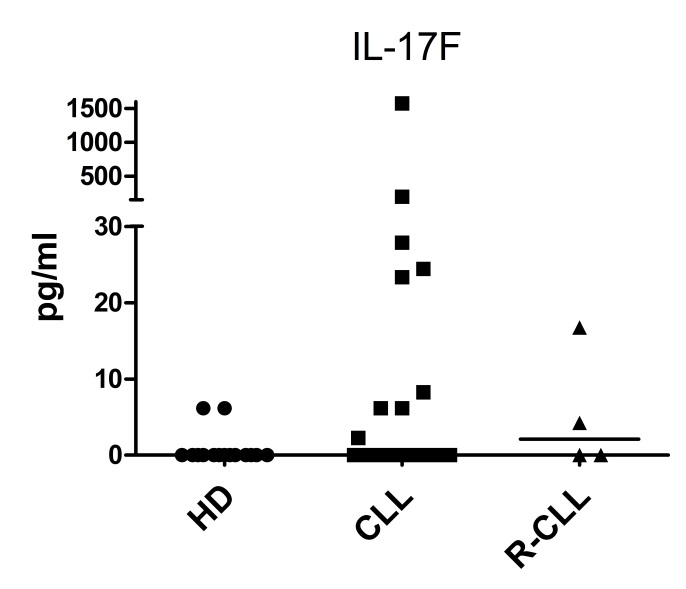


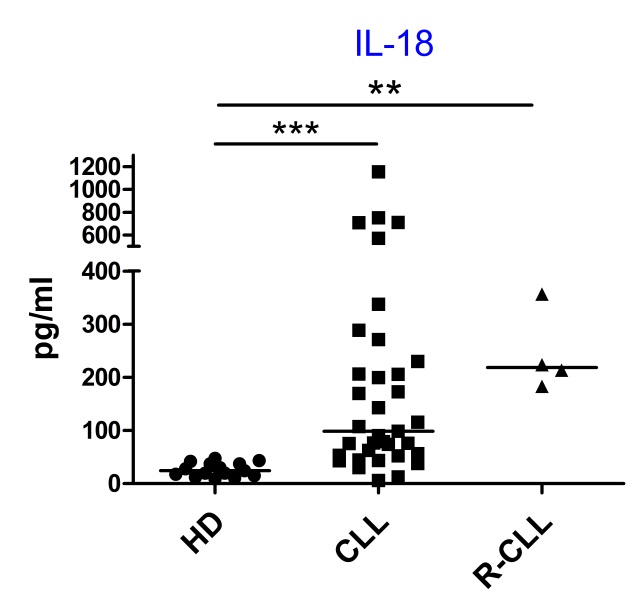

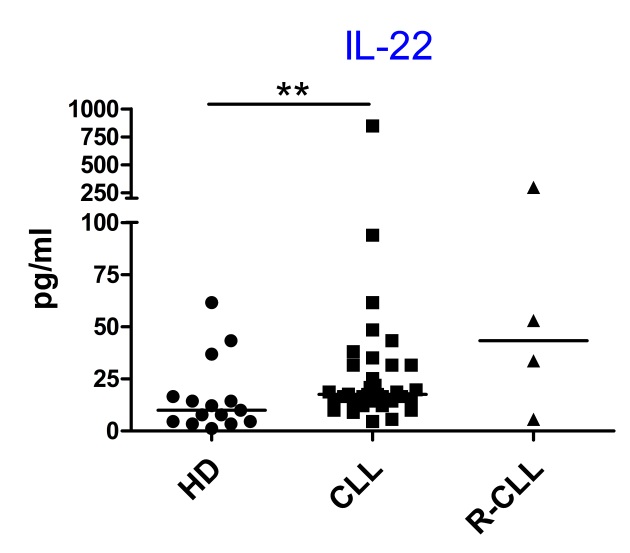


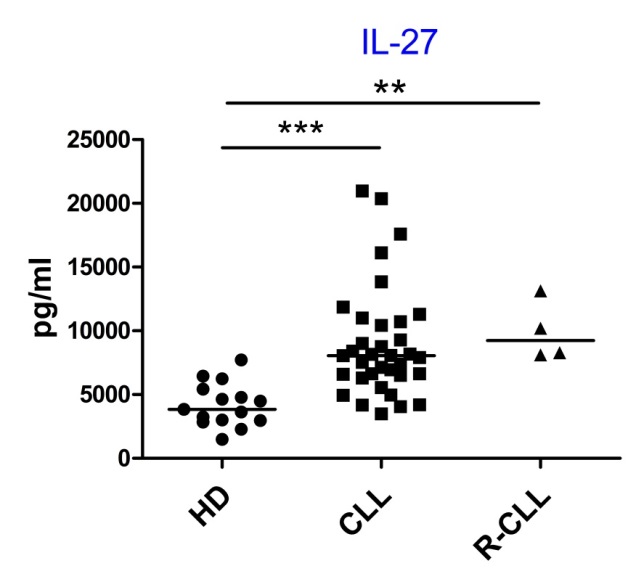

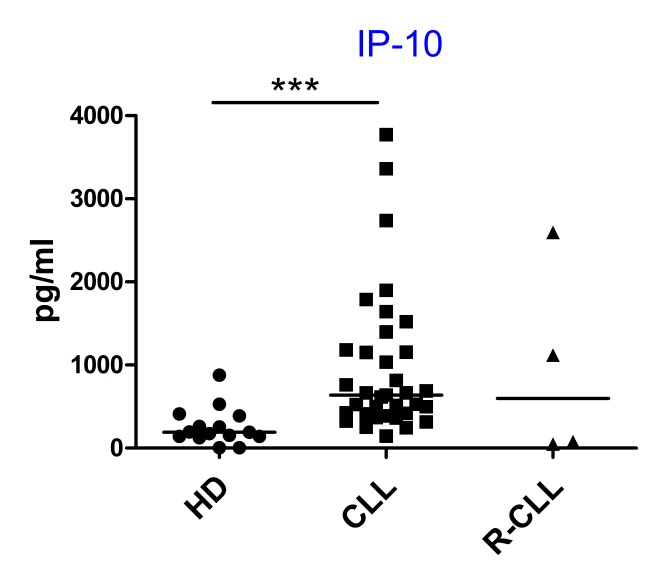


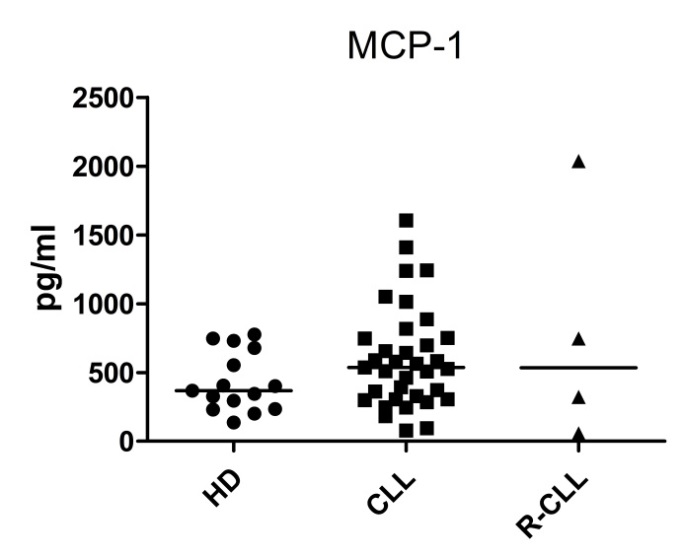

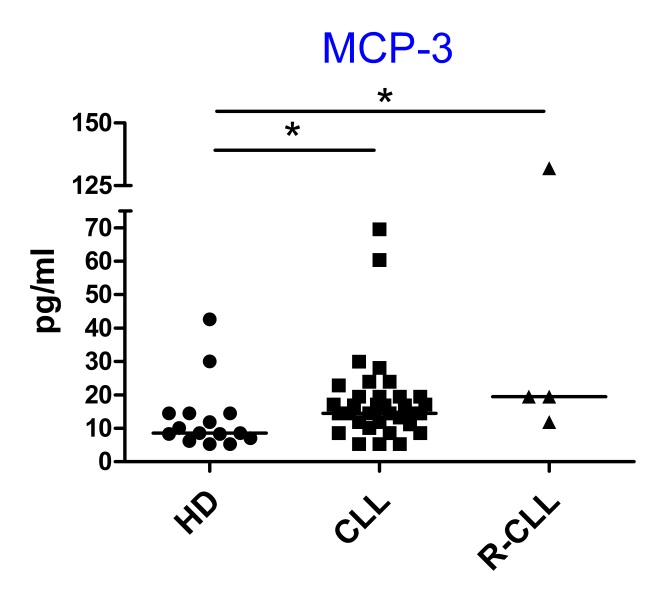


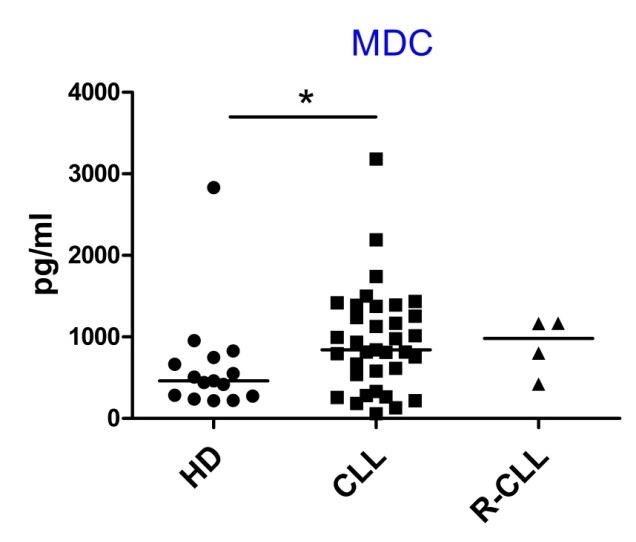

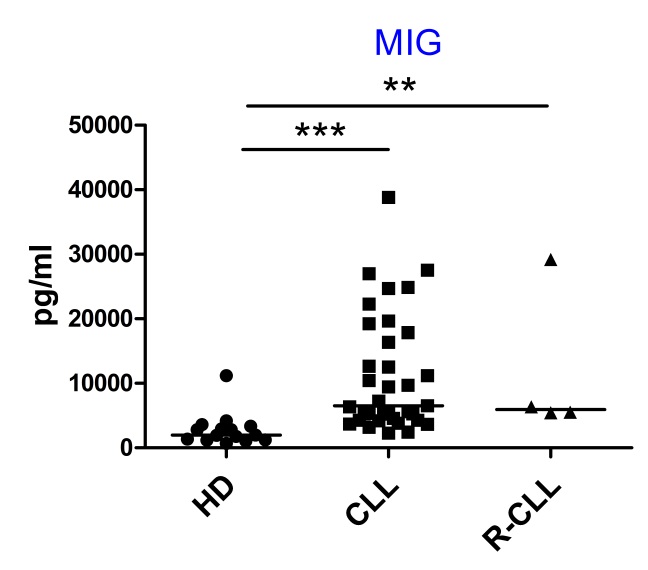


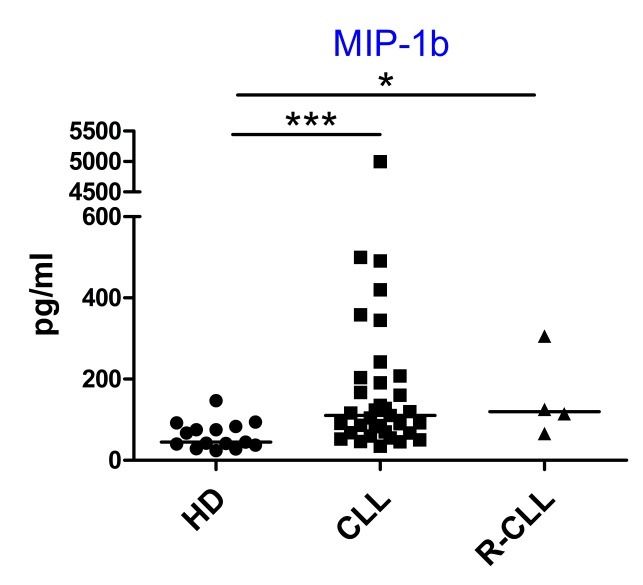

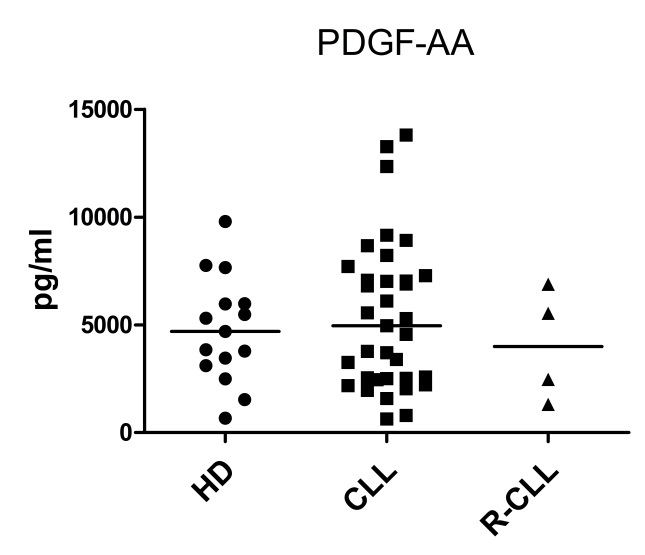


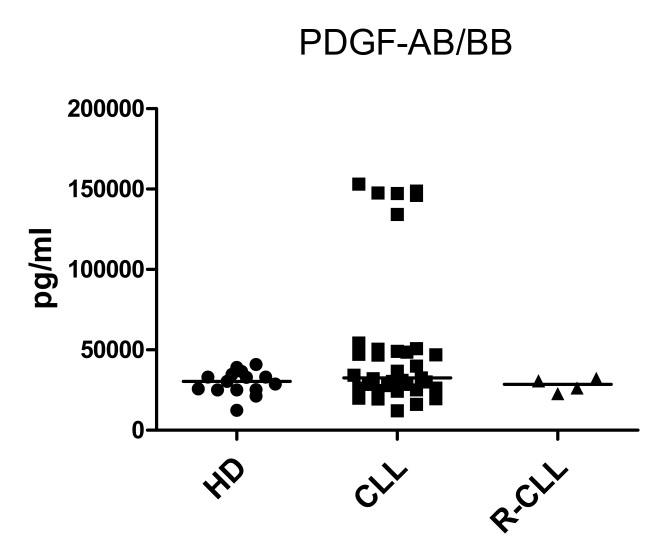

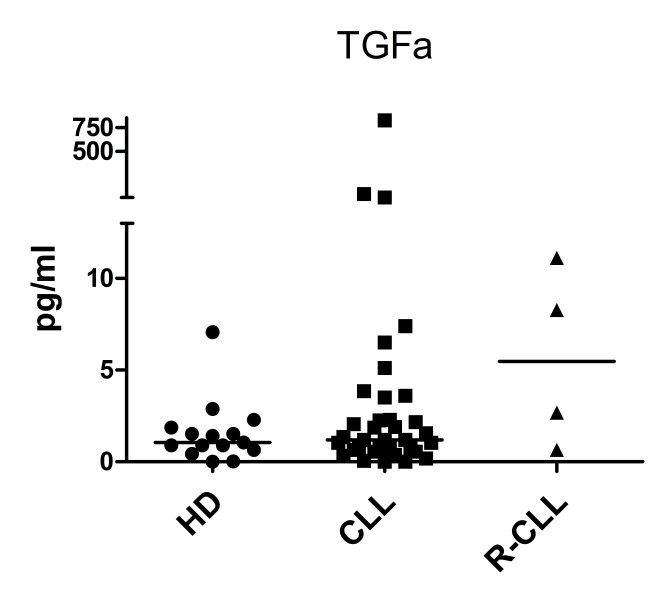


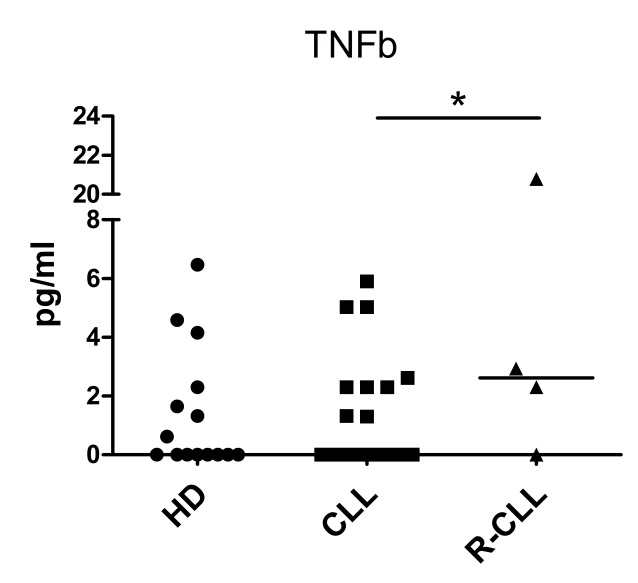

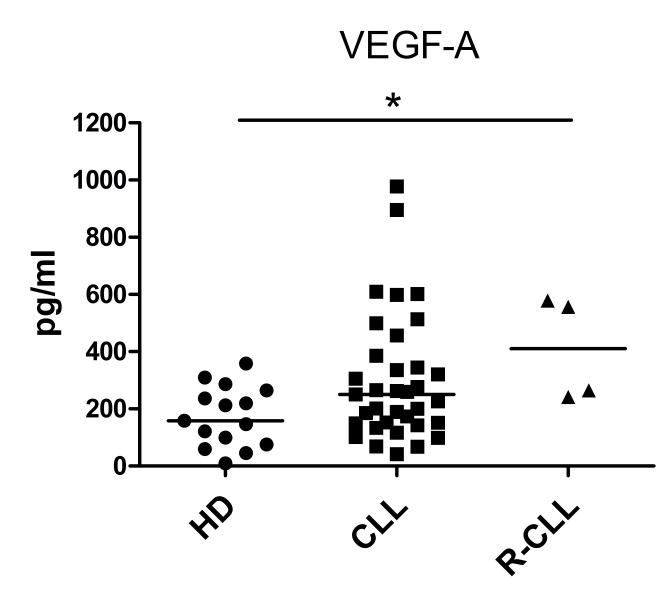

Supplement: Supplementary file 1 [file DataSheet1.docx]
